# Serving First in Isolation Increases Vegetable Intake among Elementary Schoolchildren

**DOI:** 10.1371/journal.pone.0121283

**Published:** 2015-04-01

**Authors:** Joseph P. Redden, Traci Mann, Zata Vickers, Elton Mykerezi, Marla Reicks, Stephanie Elsbernd

**Affiliations:** 1 Department of Marketing, University of Minnesota, Minneapolis, Minnesota, United States of America; 2 Department of Psychology, University of Minnesota, Minneapolis, Minnesota, United States of America; 3 Department of Food Science and Nutrition, University of Minnesota, St. Paul, Minnesota, United States of America; 4 Department of Applied Economics, University of Minnesota, St. Paul, Minnesota, United States of America; School of Public Health, Zhejiang University, CHINA

## Abstract

Many people want to eat healthier, but they often fail in these attempts. We report two field studies in an elementary school cafeteria that each demonstrate children eat more of a vegetable (carrots, broccoli) when we provide it first in isolation versus alongside other more preferred foods. We propose this healthy first approach succeeds by triggering one’s inherent motivation to eat a single food placed in front of them, and works even though they have prior knowledge of the full menu available and no real time constraints. Consistent with this theory, and counter to simple contrast effects, an additional lab study found that presenting a food first in isolation had the unique ability to increase intake whether the food was healthy (carrots) or less healthy (M&M’s). Our findings demonstrate the effectiveness of this simple intervention in promoting healthier eating, which should interest consumers, food marketers, health professionals, and policy makers.

## Introduction

Evidence for the vital importance of a healthy diet continues to accumulate, with benefits that include lower risks of obesity [1, 2], heart disease and cancer [3, 4], and the promotion of healthy aging [5]. An overall healthy diet requires not only limits on certain foods (e.g., those high in calories, fat, sugar, or salt), but also ample intake of healthy foods such as vegetables. Unfortunately, only 13% of Americans and less than 5% of children aged 9–13 eat the recommended daily amounts of vegetables [6]. This latter group is an especially crucial target because eating habits are largely formed at an early age [7], and consequences of an unhealthy diet (such as obesity) can be difficult to overcome once they manifest [8]. The present research establishes a strategy for addressing this challenge—the order of food presentation can be used to effectively increase the amount eaten of a healthy food.

A potential barrier to healthier eating is that people greatly enjoy the intrinsic attributes of sugar and fat that make less healthy foods less healthy [9]. Even before tasting a food, people often infer that it will be less tasty if it is healthy [10]. These factors make increasing vegetable intake a constant uphill battle. Consequently, many efforts to promote healthier eating through encouragement and education have proven somewhat disappointing [11–14]. We instead leverage existing psychological mechanisms to promote vegetable consumption with little effort.

When deciding what to eat, consumers often have many foods available [15], whether in their kitchens, at a buffet, or on a restaurant’s menu. Faced with such great variety, the consumer must decide which of the foods to eat on that occasion. We propose that, as with other judgments [16, 17], vegetables seem relatively unappealing when alongside a more preferred food on the same shelf, plate, or menu. This phenomenon is readily apparent by imagining the difficulty in getting a child to eat carrots when candy is present. As a result, people are unlikely to eat much of a vegetable when other better-liked foods are also readily available, which could help explain why the average person consumes less than the recommended amount of vegetables [6, 18].

We propose a simple solution to avoid the unfavorable context for vegetables: present vegetables first in isolation. When a food is presented alone, we do not expect people to spontaneously consider foods that are not present. In the context of decision making, individuals tend to base judgments largely on explicitly provided information [19, 20], and fail to spontaneously consider alternative options or opportunity costs [21]. Thus, this approach transforms a comparison between a vegetable and a more preferred food into an initial comparison between a vegetable and no food (with just the potential for other future foods). When pitted against no food, we expect people to be inherently motivated to initiate eating the vegetable (as long as they somewhat like it). Put simply, within limits, people seem to have evolved a rule of “if I see a food, then I eat it”. Of course, such a rule would have been quite adaptive in past times when food was much scarcer. In today’s world of abundance, we propose that consumers, policy makers, and marketers can still leverage this rule to make vegetables more appealing and ultimately more consumed.

Past work accords with our notion that people will spontaneously eat a food placed in front of them. Greater physical proximity and increased visibility of a food led people to eat more of it [22]. When salad likers were served salad as a first course, they ate more of it such that it satiated them on subsequent entrees [23]. People also tend to eat all of the food that they are served [24, 25]. These findings all suggest that people will eat a food presented to them if they like it. For example, the aforementioned salad study involved only salad likers who already eagerly ate such fare, making it easy to imagine increasing their consumption. The more relevant question for healthy eating is whether an intervention leads people to eat a food they neither like nor dislike, or would not typically choose to eat. Increasing the consumption of such neutral foods is more challenging, as evidenced by the finding that the well-established intervention of larger serving sizes increased consumption for only a liked fruit (applesauce), but not less-liked vegetables (broccoli and carrots) [26]. Our work examines whether mere presentation can trigger increased consumption for mildly-liked vegetables that are a staple of a healthy diet.

We predict that the motivation to eat a presented healthy food will increase when the food is presented (1) in isolation and (2) before any other foods. When there are multiple foods present, as with a typical dinner plate with an entrée and side dishes, there will be a general motivation to eat yet no clear cue which foods to eat. For vegetables, their mere presentation in a multi-food context may do little to motivate intake because people opt instead to consume more of the better-liked items. We propose also that it matters whether a vegetable is presented before or after other foods. When other foods have just been eaten, they will serve as a natural point of comparison, and there could be lingering satiety [27] that limits consumption of a subsequent food even if the latter is eaten in isolation. This leads to our predictions that, regardless of whether a food is a vegetable or something more preferred, people will eat more of it when it is presented first in isolation versus second in isolation or versus simultaneously with other foods. Three studies confirmed our predictions, established the effectiveness of a novel intervention to increase vegetable consumption, and provided insight into the contexts where it will work best.

## Cafeteria Field Study

This study had two primary objectives. First, it tested our core prediction that people eat larger quantities of a vegetable when first receiving it in isolation versus alongside other foods. We believe this strategy will prove effective for vegetables because it leverages the inherent motivation to eat a presented food yet avoids comparisons to tastier foods. Second, this study tested whether the predicted effects of food order prove successful as a dietary intervention in the natural setting of a school cafeteria. We tested whether our strategy of first serving a vegetable in isolation increases how much of this food schoolchildren eat, compared to the typical procedure of serving it at the same time as all other foods.

### Materials and Methods

This study was conducted in an elementary school with approximately 800 students in grades K-5. Approximately 75% of students in this school district belonged to racial or ethnic minorities, and 72% were eligible for free or reduced price school meals. We worked with the cafeteria staff to ensure an identical menu on both days of the study. We used raw mini carrots (identified by cafeteria staff as a mildly-liked vegetable) as our served-first vegetable, and both days occurred on a Monday. The Institutional Review Board at the University of Minnesota approved this study design that required no individual consent for the observational field study.

On the Control day (*n* = 680) students selected and ate their lunch like every other day under “normal” conditions. Prior to entering the cafeteria line, the students briefly sat at their class table until they were summoned as a group. In the line, students self-served individually pre-portioned servings of milks, fruits, and vegetables, and kitchen staff served the hot entrée (chicken tenders) and buttered noodles. At the end of the line, students were free to select second helpings of fruits and vegetables. The students then returned to the tables and ate their lunches.

On the Vegetables-First day (*n* = 755), which occurred approximately three months later when the exact same menu reappeared, our research staff prepared a small paper cup for each student that contained two raw mini carrots (the same as those available from the line). We placed these cups on the table so that there would be a cup in front of each student upon arrival. Students could eat these carrots as they waited to enter the line, but were not explicitly instructed or encouraged to eat them. Aside from the cups of carrots, the foods served and the cafeteria procedures were exactly the same as on the Control day.

After each class of students exited the lunchroom, we measured the quantity of remaining carrots by counting the uneaten carrots in each served-first cup (to the nearest half carrot) and weighing any carrots taken from the line that were still on the tray and surrounding areas. The total amount of carrots *eaten from the served-first cups* was calculated by subtracting the number of remaining carrots in each cup from the initial total amount of two, and then multiplying by the average weight of a single carrot (*M* = 10.5 g in pre-test sampling). The total amount of carrots *eaten from the serving line* was calculated by multiplying the average weight of a portion of carrots (*M* = 55.9 g) by the number of students that took carrots in the line, and then subtracting the weight of the uneaten carrots remaining on the tray. The mean amount eaten per student was calculated by dividing each of these total amounts consumed by the number of students eating the school lunch in the cafeteria, whether they took carrots or not.

### Results

Table 1 shows the mean quantity of carrots eaten on each day. When students received cups before entering the line, they ate an average of 10.1 g of carrots from the cup (*t*(755) = 6.90, *p* < .0001), which was approximately half (*M* = 48%) of the carrots in a single cup. Over 54% of the students ate at least some of the carrots from the served-first cups. This resulted in students consuming more carrots in total on the day carrots were served first in isolation versus the Control day (*M*_*Intervention*_ = 12.7 g vs. *M*_*Control*_ = 2.4 g; *t*(1433) = 14.95, *p* < .0001, *d* = .83). Even so, the likelihood of choosing carrots from the line did not differ between the two treatment days (*M*_*Intervention*_ = 9.3% vs. *M*_*Control*_ = 11.8%; χ^2^(1) = 2.37, *p* > .12), nor did the mean amount of carrots eaten from the line differ (*M*_*Intervention*_ = 2.5 g vs. *M*_*Control*_ = 2.4 g; *t* < 1, *ns*). The net result was an increase in carrot consumption of over 430% that was almost entirely driven by many students eating carrots from the cups before entering the line.

**Table 1:** Amount of Carrots Taken and Consumed per Student Eating Lunch in Cafeteria Study.

	Grams Eaten from Served-First Cups*M* (*SE*)	Number of Students Taking Carrots from Line (% of total)	Grams Eaten from Line *M* (*SE*)	Total Grams Eaten *M* (*SE*)
Control Day (*n* = 680)	---	80 (11.8)	2.39 (0.36)	2.39 (0.36)
Intervention Day (*n* = 755)	10.14 (0.38)	70 (9.3)	2.52 (0.37)	12.67 (0.57)

### Discussion

The results confirm our prediction: consumption of a vegetable increased when it was served first in isolation. We propose that this happened because our intervention kept students from choosing between a mildly-liked healthy food and more preferred foods. When given this simultaneous choice of what to eat, most schoolchildren here consistently failed to choose carrots (presumably they liked them less), while readily eating chicken tenders (casual observation that little remained). However, when given the carrots to eat first in isolation, most schoolchildren found them acceptable and willingly ate them. The schoolchildren acted as if they had a rule to eat a food presented to them, even if it was a vegetable they do not typically choose to eat.

This study provided a strong test of our theory because this population does not eat many vegetables and has generally proven resistant to interventions [14, 28, 29]. In fact, less than 12% of the children here spontaneously chose carrots from the line on the Control day, indicating that the carrots were clearly not a highly preferred food. Regardless, our intervention still proved effective in increasing participation and overall intake. It is also notable that our intervention here required very few resources (the cost of paper cups and the time to set them up). Even so, the increase in intake was comparable to other more resource-intensive efforts focused on direct encouragement through a revised school curriculum [30], contests [31, 32], or parent education events [33]. The inherent motive to eat a presented food seems to be similar in power to more conscious, effortful approaches for behavioral change.

## Longitudinal Cafeteria Field Study

This study had two primary objectives. First, if our findings are to inform a wide range of cafeterias, then we must show the effect generalizes across other settings. This study tests whether our core prediction holds specifically for a different vegetable (broccoli) and a different presentation procedure (eat while standing in the register line). Second, if our findings are to be useful for dietary change, then we must establish that our intervention remains effective over time. Schoolchildren could become accustomed to the way that foods are served to them and notice only salient changes in the process. To test for this, and rule out a simple one-time novelty effect, we expanded this study to include a baseline control day, three intervention days spread over approximately 8 weeks, and a follow-up control day.

### Materials and Methods

Participants were schoolchildren from the same elementary school (grades K-5) as the previous study. Although approximately two-thirds of the participants were enrolled during the previous study, this study occurred over two years later, ensuring that any carryover effects were quite unlikely. We again worked with the cafeteria staff to ensure the menu was identical on all days of the study. The study consisted of five Mondays spaced approximately two to three weeks apart, which was how frequently the same daily menu was served. There was an initial baseline Control day, followed by three intervention Vegetables-First days, and concluding with a final Control day. The Institutional Review Board at the University of Minnesota approved this study design that required no individual consent for the observational field study.

Students were escorted to the lunch line by their teacher in one of nine lunch periods. On the initial and follow-up Control days (*n* = 558 and *n* = 529 respectively), students served themselves vegetables and fruits from a buffet-type line, while a cafeteria employee served the hot side (vegetable fried rice) and entrée (teriyaki chicken). We specifically tracked taking from the line and consumption of broccoli and cauliflower, each pre-portioned in four-oz. (120 mL) cups. We included two vegetables because while the focus of our intervention was broccoli consumption, we also needed a control vegetable (cauliflower) to test whether increased consumption of broccoli largely came at the expense of cauliflower consumption.

On the three Vegetable-First days (*n* = 486, *n* = 530, and *n* = 529 respectively), our research staff prepared a small serving of broccoli (2 pieces) in two-oz. paper cups. These cups were handed to each student in the hallway before entering the lunch line, and importantly there was no verbal encouragement to eat them. This allowed the students a few minutes to eat their broccoli while waiting in line to enter their ID numbers at a register. Aside from receiving the cup of broccoli, the foods served and the cafeteria procedures were identical to the Control days.

After each class of students finished their meal, a trained member of the research team visually assessed and recorded how much was left in each of the broccoli cups (to the nearest whole number). The total amount of broccoli *eaten from the served-first cups* was calculated by subtracting the number of remaining broccoli pieces in each cup from the initial total amount of two, and then multiplying by the average weight of a single broccoli piece. The average weight varied across the three intervention days (*M* = 5.3, 4.5, and 4.3 g respectively in pre-test sampling) due to natural variations in what the supplier provided. Given that the quantity decreased over time, any portion size effects would have worked against our intervention holding up over time. We also collected all broccoli waste from the cafeteria entrance area, and allocated the waste to each student as a proportion of how much they had eaten. The total amount of a vegetable *eaten from the serving line* was calculated by multiplying the number of pieces in a portion (8 pieces) by the number of students that took that vegetable in the line, and then subtracting the weight of that uneaten vegetable remaining on the tray. The mean amount eaten per student was calculated by dividing each of these total amounts consumed by the number of students eating the school lunch in the cafeteria, whether they took a vegetable or not.

### Results

#### Broccoli total consumption

Table 2 reports the mean quantity of broccoli eaten on each day. We analyzed the total quantity consumed per student using a mixed model with the day and grade level (and their interaction) as fixed effects, and the student-level intercept as a random effect. We included the grade factor because past work has found that children develop a growing understanding of persuasion and conformity during the elementary school years [34, 35]; however, we had no specific predictions in regards to age. The results indicated a main effect of day (*F*(4, 613) = 72.60, *p* < .0001), a marginal main effect of grade (*F*(5, 613) = 2.22, *p* < .06), and an interaction (*F*(20, 613) = 1.96, *p* < .01).

**Table 2:** Amount of Broccoli Taken and Consumed per Student Eating Lunch in Longitudinal Study.

	Grams Eaten from Served-First Cups*M* (*SE*)	Number of Students Taking Broccoli from Line (% of total)	Grams Eaten from Line *M* (*SE*)	Total Grams Eaten *M* (*SE*)
Initial Control Day (*n* = 558)	---	77 (13.8)	0.84 (.16)	0.84 (.16)
Intervention Day 1 (*n* = 486)	3.43 (.21)	20 (4.1)	0.57 (.14)	3.99 (.25)
Intervention Day 2 (*n* = 530)	2.53 (.15)	50 (9.4)	1.52 (.22)	4.06 (.27)
Intervention Day 3 (*n* = 529)	1.53 (.10)	28 (5.3)	0.57 (.12)	2.10 (.16)
Final Control Day (*n* = 529)	---	45 (8.5)	0.90 (.16)	0.90 (.16)

Planned contrasts uncovered the nature of the effects and tested our specific predictions. Consistent with the previous study, students consumed more broccoli on the first intervention day (*M* = 3.99 g) than either the initial control day (*M* = .84 g; *t*(613) = 12.14, *p* < .0001, *d* = .67) or final control day (*M* = .90 g; *t*(613) = 12.14, *p* < .0001, *d* = .67). The effectiveness of the intervention also persisted over time as this result held true for the second intervention day (*M* = 4.06 g; contrasts with control days both had *p* < .0001 and *d* > .62), and the third intervention day (*M* = 2.10 g; contrasts with control days both had *p* < .0001 and *d* > .32). Although there was no difference in broccoli consumption between the first two intervention days (*t* < 1, *ns*), consumption was lower on the third day than both the first day (*t*(613) = 7.42, *p* < .0001, *d* = .40) and the second day (*t*(613) = 7.48, *p* < .0001, *d* = .38). There were no apparent lingering effects of the intervention as the initial and final control days showed no difference (*t* < 1, *ns*).

#### Broccoli served-first cup and line consumption

We next analyzed how each of the sources of the vegetable (cup or line) contributed to total consumption. On the three intervention days, students ate 3.43, 2.53, and 1.53 g respectively of the broccoli in the served-first cup. This represented 34%, 28%, and 19% respectively of the total amount in the cup. Although they ate less than half of the broccoli in the cup, this still represented 85%, 62%, and 73% of the total broccoli consumption respectively for each day.

We also analyzed the effect of providing a served-first cup of broccoli on the choice and consumption of broccoli from the line. The percentage of students taking broccoli from the line was higher on the initial control day (13.8%) than the first (4.1%; χ^2^(1) = 26.06, *p* < .0001), second (9.4%; χ^2^(1) = 8.58, *p* < .01), and third intervention days (5.3%; χ^2^(1) = 21.70, *p* < .0001). Similarly, the choice percentage was higher on the final control day (8.5%) than the first intervention day (χ^2^(1) = 9.31, *p* < .01), and third intervention day (χ^2^(1) = 7.31, *p* < .01), but not the second intervention day (χ^2^ < 1, *ns*). This pattern of results suggests that the intervention potentially cannibalized somewhat from the line. However, the extent of cannibalization was minimal as the average quantity eaten from the line was not significantly higher on the initial control day (*M* = .84 g) than any of the intervention days (*M* = .57, 1.52, and .57 g respectively, all *p* > .11). This indicates that consumption from the cup tended to lower choice from the line primarily for those who tended not to eat what they chose from the line anyway. The net result was an increase in broccoli consumption on the intervention days ranging from 150% to over 380% that was almost exclusively linked to consumption of the broccoli from the cups.

#### Intervention effects by grade

Given there was an intervention day by grade effect, we first confirmed that our results were not limited to particular grades. When we collapsed total broccoli consumption together for the control and intervention days respectively, the contrast between these two was statistically significant at each of the six grade levels (all contrasts have *p* < .001). This indicates that our intervention was effective regardless of the grade. In fact, although there was a significant interaction, the size of the control versus intervention contrast did not significantly differ between any of the grade pairs (all *p* > .06). There was also no obvious trend as the size of this contrast was 2.30, 2.59, 2.59, 1.84, 2.75, and 3.00 g for each respective grade. These findings all suggest that the intervention successfully increased broccoli consumption regardless of the age of the child.

#### Cauliflower total consumption

Table 3 reports the mean quantity of cauliflower eaten on each day. We analyzed the total quantity consumed per student using the same mixed model as for the broccoli. The results indicated a main effect of intervention day (*F*(4, 613) = 2.96, *p* < .02), a marginal main effect of grade (*F*(5, 613) = 2.18, *p* < .06), and a non-significant interaction (*F*(20, 613) = 1.02, *p* > .43). Further contrasts did not reveal an obvious effect of the intervention days versus the control days. If anything, the initial control day tended to have less cauliflower consumption (*M* = .57 g) than the intervention days, but this difference was statistically significant for only the third intervention day (*M* = 1.33 g; *t*(613) = 2.50, *p* < .02, *d* = .15). In contrast, the final control day tended to have greater cauliflower consumption (*M* = 1.35 g) than the intervention days, but this difference was significant for only the second intervention day (*M* = .57 g; *t*(613) = 2.33, *p* < .03, *d* = .15). Overall, the intervention with the cups of broccoli did not appear to have a systematic effect on the consumption of cauliflower.

**Table 3:** Amount of Cauliflower Taken and Consumed per Student Eating Lunch in Longitudinal Study.

	Students Taking Cauliflower from Line *n* (% of total)	Grams Eaten from Line *M* (*SE*)
Initial Control Day (*n* = 558)	22 (3.9)	0.57 (.17)
Intervention Day 1 (*n* = 486)	22 (4.5)	0.88 (.22)
Intervention Day 2 (*n* = 530)	15 (2.8)	0.57 (.17)
Intervention Day 3 (*n* = 529)	29 (5.5)	1.33 (.26)
Final Control Day (*n* = 529)	27 (5.1)	1.35 (.27)

### Discussion

We again confirmed our predictions that serving a vegetable first in isolation can increase its consumption in an elementary school cafeteria. We replicated this result using a different vegetable (broccoli), which suggests that our intervention would be effective across a wide range of vegetables. We also generalized our result by using a different procedure for delivering the vegetable first. Here, rather than having students sit down with cups, we provided students the cups while they waited in line. This procedure should be fairly easy to implement in almost any school cafeteria, again suggesting the intervention could prove successful in a wide range of settings.

We also examined the broader long-term effects of our intervention. In particular, we confirmed that the intervention maintained its effectiveness even with repeated exposures. This indicates that our effects are not simply due to novelty. The repeated effectiveness also establishes that the success of our intervention is not solely rooted in initiating trial of a food the children will just learn they do not like anyway. Our intervention showed no loss of effectiveness on the second day, indicating that our results are not driven by a one-time trial. Rather, our intervention remained effective (versus the control days) even after multiple exposures, which is an important consideration frequently ignored in past work on healthy eating interventions [36]. Here, we found that the intervention showed no decline in effectiveness on the second exposure, but a drop did emerge on the third exposure. However, the third exposure was still clearly effective at increasing intake versus the control days.

We also confirmed that although our intervention increased broccoli consumption, it did not decrease consumption of another vegetable (cauliflower). This indicates our intervention is likely to increase overall vegetable consumption, rather than cannibalize other vegetables. It is notable that we also found no lingering effects of our intervention on vegetable consumption once we ceased presenting them first in isolation. This shows that cafeterias can continually benefit from our intervention strategy, but it requires a permanent change to cafeteria procedures.

## Lab Study

This final follow-up lab study had three primary objectives. First, it tested whether our predicted effects of food order hold in a more focused lab setting with adults. Although the lab setting encourages greater attention to eating, we still expected that serving a vegetable first in isolation would increase intake versus serving it alongside other better-liked foods. Second, this study also tested our prediction for a more preferred food to determine which of two underlying mechanisms drives our findings. If the desire to eat any presented food dominates, as we propose, then eating a preferred food first in isolation should increase intake as it does for a less preferred vegetable. Alternatively, it could be that eating a food first lessens comparisons to other foods [16, 17]. If such contrast effects dominate, then eating a preferred food in isolation should decrease its intake (as this food will not get the liking boost that would come from the favorable comparison to a less-liked food). Therefore, if serving a well-liked food first still increases intake of that food, then our account is supported and the contrast explanation is contradicted. Third, this study measured the intake of every food offered, which was logistically infeasible in the cafeteria setting. This provided a more complete gauge of the extent to which satiety could account for our findings. If satiety largely drives the effect, then participants should eat a larger amount of the first food presented and less of any subsequent food. Such satiation would then predict that participants will eat much less of a food when it is presented second after another food versus simultaneously with the other food. If we do not find this pattern of results, then satiation is unlikely to fully account for our results, and we will have support for our theory.

### Materials and Method

One hundred and eighteen people (69 females, 46 males, 3 unknown; *M*_*Age*_ = 23) participated in exchange for $6 at a university research lab. The Institutional Review Board at the University of Minnesota approved the study design that began with written consent. We manipulated the snack type as a within-subjects factor with the two levels of Less-liked (Earthbound Farm Organic Mini Peeled Baby Carrots) and More-liked (M&M’s). We chose these two particular snacks because they are familiar to nearly everyone, and the suggested serving size of the single-serving snack packages of each was approximately the same number of grams. We had also pre-tested these snacks on 0 to 10 scales to confirm that our population perceived carrots as less unhealthy (*M*_*Carrots*_ = 0.37 vs. *M*_*M&M’s*_ = 8.12; *t*(66) = 28.17, *p* < .0001), and less liked (*M*_*Carrots*_ = 6.64 vs. *M*_*M&M’s*_ = 7.64; *t*(66) = 2.15, *p* < .04).

Each participant was presented a total of 50 g of each snack (approximately the size of the single-serving snack package for each snack), and allowed to freely eat it while watching two five-minute animated videos. We manipulated the order of snack presentation by randomly assigning each session (up to six participants each in a separate room) to one of only three conditions. Participants in the Simultaneous condition (*n* = 39) received 50 g of each snack in two bowls right before the videos started playing. Participants in the Less-liked (Carrots) First condition (*n* = 36) first received 50 g of carrots in a bowl, and were told “Here are the carrots, and we also have M&M’s. I still have to get them for you. I’ll be back in about five minutes”. These instructions ensured all participants realized that they would have the chance to eat both snacks. After five minutes, these participants received 50 g of M&M’s in a separate bowl. Participants in the More-liked (M&M’s) First condition (*n* = 43) followed the same procedure except they first received 50 g of M&M’s, and then received a bowl with 50 g of carrots after five minutes. After participants had finished watching both videos and had exited the lab, we weighed the remaining quantity of each snack to gauge the quantity consumed.

### Results

**Fig 1:**
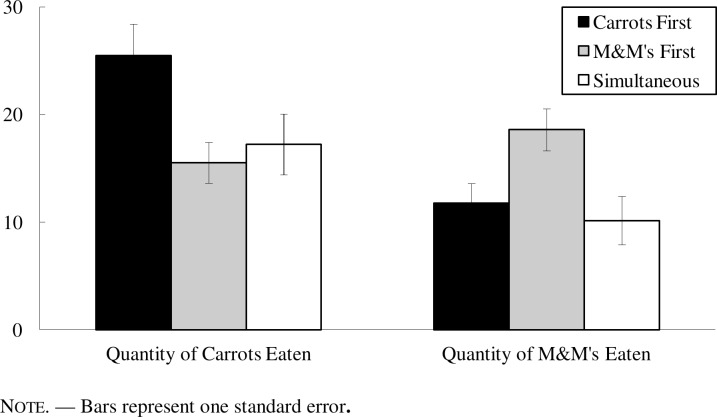
Grams of Each Snack Eaten by Condition in Lab Study.

Fig. 1 reports the mean quantity eaten by condition. We tested for differences in these means using a repeated-measures ANOVA on the intake in grams for each snack with snack type as a within-subjects factor and presentation order as a between-subjects factor. There was evidence of a main effect for snack type such that more carrots were eaten than M&M’s (*F*(1, 115) = 11.82, *p* < .001, η_*p*_^*2*^ = .09), but no main effect for presentation order (*F*(2, 115) = 2.00, *p* > .14). More importantly, these effects were qualified by the presence of the key two-way interaction (*F*(2, 115) = 8.20, *p* < .001, η_*p*_^*2*^ = .12). As we predicted, each snack was eaten more when presented first in isolation.

Given the presence of the interaction, we ran separate one-way ANOVAs for each snack type to further test our predictions. For intake of the less-liked snack of carrots, we confirmed the effect of presentation order (*F*(2, 115) = 4.20, *p* < .02, η_*p*_^*2*^ = .07). Participants first eating carrots ate more carrots than did those eating the more-liked M&M’s first (*M*_*CarrotsFirst*_ = 25.5 g vs. *M*_*M&MsFirst*_ = 15.5 g; *t*(115) = 2.75, *p* < .01, *d* = .66), or those eating the two snacks simultaneously (*M*_*CarrotsFirst*_ = 25.5 g vs. *M*_*Simultaneous*_ = 17.2 g; *t*(115) = 2.23, *p* < .03, *d* = .47). The results for the intake of M&M’s followed the same pattern. There was an effect of presentation order (*F*(2, 115) = 5.20, *p* < .01, η_*p*_^*2*^ = .08), and consumption of M&M’s was greatest when eaten first in isolation versus eaten second (*M*_*M&MsFirst*_ = 18.6 g vs. *M*_*CarrotsFirst*_ = 11.8 g; *t*(115) = 2.39, *p* < .02, *d* = .58), or simultaneously with the carrots (*M*_*M&MsFirst*_ = 18.6 g. vs. *M*_*Simultaneous*_ = 10.2 g; *t*(115) = 3.03, *p* < .01, *d* = .63). Across the analyses, regardless of the snack, we consistently found that eating a snack first in isolation increased its intake versus the other conditions.

We also analyzed the intake for each snack when it was presented second versus simultaneously with the other snack. Participants ate a statistically identical amount of carrots whether they were presented second or simultaneously (*M*_*Second*_ = 15.5 g vs. *M*_*Simultaneous*_ = 17.2 g; *t* < 1, *ns*), and likewise for M&M’s (*M*_*Second*_ = 11.8 g vs. *M*_*Simultaneous*_ = 10.1 g; *t* < 1, *ns*). This indicates that our findings are likely not due to satiation, and that our effects on the intake of a food are unique to the case in which that food was presented first in isolation.

### Discussion

Although practical considerations prevented us from bringing elementary schoolchildren into the lab, which limits inferences for that population, we again found clear evidence that serving a food first in isolation increases its intake. The effects on eating behavior extended to an undergraduate population were not trivial as intake increased by over 56% for carrots and 70% for M&M’s compared to the groups not eating that snack first. These findings provide strong evidence that eating a snack in isolation can be an effective strategy to encourage eating that food, even though everyone had full information about all upcoming foods. In fact, even though participants here ate a fair amount of the carrots in every condition (at least 30% of what they were given on average), the intervention was still able to substantially increase the consumption of this vegetable. We expect that this strategy will prove even more powerful when a food is not naturally chosen very often (as in the previous cafeteria studies).

The pattern of the results in this study also helps rule out some alternative explanations. It could be that participants ate more of the food presented first simply because it was available for a longer period of time. However, if exposure time drove our effects then participants should have eaten the most in total when both snacks were available for the entire study, yet we find the opposite pattern if anything (*M*_*Simultaneous*_ = 27.4 g vs. *M*_*Not Simultaneous*_ = 35.6 g; *t*(116) = 1.90, *p* < .06). There is also evidence that our findings do not simply reflect satiation from eating the first food. Specifically, participants ate the same amount of a snack whether it was provided simultaneously or after another snack. Increased satiation from eating the first snack should lead to a decreased intake of the snack presented second, yet we found little evidence for this. Of course, with much larger servings, satiation could become an influential factor.

The results also speak to whether the increased consumption of a food presented first in isolation is largely driven by contrast effects. If the contrast in liking was the key driver, then eating a better-liked food in isolation should decrease its intake as it no longer benefits from a favorable liking contrast with a less-liked food. Our results also did not support this story as eating better-liked M&M’s first increased M&M intake by over 70% versus not eating them first, suggesting M&M intake benefited little from a comparison with the less-liked carrots. Alternatively, we attribute the findings to an inherent motivation to eat a food when presented, which seems to operate primarily when there is only a single food present. Our proposed theory then easily accounts for the full pattern of findings (and the previous two cafeteria studies).

## Conclusions

The United States is fighting an ongoing battle to improve its health, with 35% of adults considered obese and an additional 34% considered overweight [37]. A popular recommendation for better health is greater consumption of fruits and vegetables [38–40], especially among young children. Unfortunately, past efforts to increase vegetable intake have been largely disappointing [11, 13, 14, 29]. Our work provides an explanation for why many past interventions may have had little effect: vegetables are usually presented alongside better-liked foods. This salient disadvantage in liking is then difficult to overcome with interventions that are based solely on education and raising awareness. Put simply, it is difficult to get a person to eat something they only slightly like when more preferred foods are also available.

Our findings suggest that a more effective strategy is instead to serve the vegetables first in isolation. Three experiments established that serving carrots and broccoli first in isolation successfully increased consumption of these foods. This approach leverages a rule that people seem to follow: eat a single food that is in front of you. In some ways, this behavior seems fairly intuitive, but in other ways there is nothing intuitive about getting a child to eat a vegetable they find barely acceptable. Our findings are indeed quite inconsistent with a fully rational decision maker who, knowing they will have other foods in the future, can just choose to skip whatever food is presented first and eat only the foods they like the most. Of course, presentation order of a food is not the only consideration, as people may still rely on their beliefs about the foods, dieting goals, or the behavior of those around them. Even so, we found a consistent effect of presentation order in our studies. Future work can still explore how the relative contribution of other factors may depend on the context.

Our work contributes to the literature in several important ways. We provide a novel low-cost approach to increase vegetable consumption among the notoriously challenging population of elementary school children. This group chronically under-consumes vegetables and has proven resistant to many interventions with the goal to increase vegetable consumption [14, 29, 41]. We expect that the strength of our results coupled with the ease of serving vegetables first make our intervention applicable to a wide range of settings (most cafeterias, restaurants, or meals in the home). Our findings also generalize past work on eating vegetables first [23] in several critical ways. We show that serving a food first can increase intake for a less-liked food (carrots and broccoli), versus just well-liked foods, which is essential for promoting the consumption of vegetables. We also show our effects persist even with repeated exposures spanning nearly two months, and with children not actively supervised by a parent or guardian (an understudied setting). Finally, we also combine our field data with a lab study to provide support for our proposed theory over alternative accounts based in contrast or satiety effects. Overall, we establish an intervention to promote vegetable consumption, demonstrate its sizable effect, show its robust nature, and provide insight into the conditions that promote it. The effectiveness of our intervention is notable in that it produced sizable effects with very little effort, and appeared robust to different foods and even repeated exposures. This resembles other approaches rooted in behavioral economics such as suggesting norms [42], redesigning cafeteria processes [43], and offers to downsize [44]. In our first cafeteria study, merely placing cups of carrots on the table before students arrived increased carrot consumption by more than a factor of four. The net result was an increase in carrot consumption of 10.3 g, or .13 portions, which favorably compares to the average increase of .07 portions in vegetable consumption found in a recent meta-analysis for interventions targeted at children aged 9 to 12 years [41]. This suggests that school cafeterias, as well as parents, not serving vegetables first in isolation (which is likely true for most cafeterias and homes) could benefit from our findings.

Given the effectiveness of our intervention relative to its implementation cost, future work should test our simple intervention across a range of settings. This should include cafeterias with a range of mealtime procedures so our intervention can apply as widely as possible. We have successfully implemented two different procedures in our field studies, but others are possible. The logistics of serving vegetables first may prove particularly challenging to commercial food service establishments. We also confirmed that our intervention remained effective over three repeated exposures, but had few lingering effects once removed. This suggests that cafeterias should consider making serving vegetables first in isolation a permanent part of their procedures. Of course, further investigation with longitudinal field experiments are still necessary to shed light on the long-term effectiveness of this intervention strategy. As well, future work should examine how eating more vegetables influences future consumption. If our intervention indeed reduces subsequent intake of less healthy foods, as we expect it might, then it could prove quite effective at improving one’s diet.

We believe that eating vegetables first in isolation may prove useful to a wide range of audiences that include children, dieters, parents, school officials, and public policy makers. Parents, for example, might increase vegetable consumption by simply serving vegetables as an appetizer before serving the rest of the meal. Future research could also test whether our intervention encourages habit formation. Past work has shown it takes multiple tastes to develop a liking for a food [45], so any instance of eating vegetables may increase the likelihood of eating it again in the future. Regardless, we propose that by isolating vegetables as a first course, those trying to increase vegetable consumption have a greater likelihood of immediate success.

## References

[1] Tande DL, Magel R, Strand BN. Healthy Eating Index and abdominal obesity. Public Health Nutrition 2010;13(02): 208–214. 19650960 10.1017/S1368980009990723

[2] Gao SK, Beresford SA, Frank LL, Schreiner PJ, Burke GL, Fitzpatrick AL. Modifications to the Healthy Eating Index and Its Ability to Predict Obesity: the Multi-Ethnic Study of Atherosclerosis. The American Journal of Clinical Nutrition 2008;88(1): 64–69. 18614725 10.1093/ajcn/88.1.64

[3] Reedy J, Krebs-Smith SM, Miller PE, Liese AD, Kahle LL, Park Y, et al Higher Diet Quality Is Associated with Decreased Risk of All-Cause, Cardiovascular Disease, and Cancer Mortality among Older Adults. The Journal of Nutrition 2014;144(6): 881–889. 10.3945/jn.113.189407 24572039 PMC4018951

[4] Kant AK, Graubard BI. A Comparison of Three Dietary Pattern Indexes for Predicting Biomarkers of Diet and Disease. Journal of the American College of Nutrition 2005;24(4): 294–303. 16093407 10.1080/07315724.2005.10719477

[5] Samieri C, Sun Q, Townsend MK, Chiuve SE, Okereke OI, Willett WC, et al The Association Between Dietary Patterns at Midlife and Health in Aging: An Observational Study. Annals of Internal Medicine 2013;159(9): 584–591. 10.7326/0003-4819-159-9-201311050-00004 24189593 PMC4193807

[6] Krebs-Smith SM, Guenther PM, Subar AF, Kirkpatrick SI, Dodd KW. Americans Do Not Meet Federal Dietary Recommendations. The Journal of Nutrition 2010;140(10): 1832–1838. 10.3945/jn.110.124826 20702750 PMC2937576

[7] Mikkila V, Rasaneu L, Raitakari OT, Pietinen P, Viikari J. Consistent dietary patterns identified from childhood to adulthood: The Cardiovascular Risk in Young Finns Study. British Journal of Nutrition 2005;93(06): 923–931. 16022763 10.1079/bjn20051418

[8] de Mutsert R, Sun Q, Willett WC, Hu FB, van Dam RM. Overweight in Early Adulthood, Adult Weight Change, and Risk of Type 2 Diabetes, Cardiovascular Diseases, and Certain Cancers in Men: a Cohort Study. American Journal of Epidemiology 2014;179(11): 1353–1365. 10.1093/aje/kwu052 24786797 PMC4036209

[9] Hoppert K, Mai R, Zahn S, Hoffmann S, Rohm H. Integrating sensory evaluation in adaptive conjoint analysis to elaborate the conflicting influence of intrinsic and extrinsic attributes on food choice. Appetite 2012;59(3): 949–955. 10.1016/j.appet.2012.09.005 23000276

[10] Raghunathan R, Naylor RW, Hoyer WD. The Unhealthy = Tasty Intuition and Its Effects on Taste Inferences, Enjoyment, and Choice of Food Products. Journal of Marketing 2006;70(4): 170–184.

[11] Knai C, Pomerleau J, Lock K, McKee M. Getting children to eat more fruit and vegetables: A systematic review. Preventive Medicine 2006;42(2): 85–95. 16375956 10.1016/j.ypmed.2005.11.012

[12] Maes L, Van Cauwenberghe E, Van Lippevelde W, Spittaels H, De Pauw E, Oppert J-M, et al Effectiveness of workplace interventions in Europe promoting healthy eating: a systematic review. The European Journal of Public Health 2012;22(5): 677–683. 21785115 10.1093/eurpub/ckr098

[13] French SA, Stables G. Environmental interventions to promote vegetable and fruit consumption among youth in school settings. Preventive Medicine 2003;37(6): 593–610. 14636793 10.1016/j.ypmed.2003.09.007

[14] Blanchette L, Brug J. Determinants of fruit and vegetable consumption among 6–12-year-old children and effective interventions to increase consumption. Journal of Human Nutrition and Dietetics 2005;18(6): 431–443. 16351702 10.1111/j.1365-277X.2005.00648.x

[15] Stevens J, Bryant M, Wang L, Borja J, Bentley ME. Exhaustive measurement of food items in the home using a universal product code scanner. Public Health Nutrition 2011;14(02): 314–318. 20602866 10.1017/S1368980010001837 PMC3126097

[16] Helson H. Adaptation-Level Theory New York: Harper & Row; 1964.

[17] Parducci A. Happiness, Pleasure, and Judgment: The Contextual Theory and Its Applications Mahwah, NJ: Lawrence Erlbaum Associates; 1995.

[18] Kimmons J, Gillespie C, Seymour J, Serdula M, Blanck HM. Fruit and vegetable intake among adolescents and adults in the United States: percentage meeting individualized recommendations. Medscape Journal of Medicine 2009;11(1): 26 19295947 PMC2654704

[19] Slovic P. From Shakespeare to Simon: Speculations—and Some Evidence—about Man’s Ability to Process Information. Oregon Research Institute Research Monograph 1972;12: 10–23.

[20] Kahneman D, Frederick SF. Representativeness Revisited: Attribute Substitution in Intuitive Judgment In: Gilovich T, Griffin D, Kahneman D, editors. Heuristics and Biases: The Psychology of Intuitive Judgment. New York: Cambridge University Press; 2002 p. 48–81.

[21] Frederick S, Novemsky N, Wang J, Dhar R, Nowlis S. Opportunity Cost Neglect. Journal of Consumer Research 2009;36(4): 553–561.

[22] Wansink B. Environmental Factors That Unknowingly Increase Food Intake and Consumption. Annual Review of Nutrition 2004;24: 341–378. 10.1146/annurev.nutr.24.012003.132140 15189128

[23] Roe LS, Meengs JS, Rolls BJ. Salad and satiety. The effect of timing of salad consumption on meal energy intake. Appetite 2012;58(1): 242–248. 10.1016/j.appet.2011.10.003 22008705 PMC3264798

[24] Siegel PS. The completion compulsion in human eating. Psychological Reports 1957;3: 15–16.

[25] Geier AB, Rozin P, Doros G. Unit Bias: A New Heuristic That Helps Explain the Effect of Portion Size on Food Intake. Psychological Science 2006;17(6): 521–525. 16771803 10.1111/j.1467-9280.2006.01738.x

[26] Kral TVE, Kabay AC, Roe LS, Rolls BJ. Effects of Doubling the Portion Size of Fruit and Vegetable Side Dishes on Children's Intake at a Meal. Obesity 2010;18(3): 521–527. 10.1038/oby.2009.243 19680238

[27] Cabanac M. Physiological Role of Pleasure. Science 1971;173(4002): 1103–1107. 5098954 10.1126/science.173.4002.1103

[28] Condon EM, Crepinsek MK, Fox MK. School meals: Types of foods offered to and consumed by children at lunch and breakfast. Journal of the American Dietetic Association 2009;109(2): S67–78. 10.1016/j.jada.2008.10.062 19166674

[29] Thomson CA, Ravia J. A Systematic Review of Behavioral Interventions to Promote Intake of Fruit and Vegetables. Journal of the American Dietetic Association 2011;111(10): 1523–1535. 10.1016/j.jada.2011.07.013 21963019

[30] Davis M, Baranowski T, Resnicow K, Baranowski J, Doyle C, Smith M, et al Gimme 5 Fruit and Vegetables for Fun and Health: Process Evaluation. Health Education & Behavior 2000;27(2): 167–176. 10768798 10.1177/109019810002700203

[31] Raju S, Rajagopal P, Gilbride TJ. Marketing Healthful Eating to Children: The Effectiveness of Incentives, Pledges, and Competitions. Journal of Marketing 2010;74(3): 93–106.

[32] Baranowski T, Baranowski J, Cullen KW, deMoor C, Rittenberry L, Hebert D, et al 5 A Day Achievement Badge for African-American Boy Scouts: Pilot Outcome Results. Preventive Medicine 2002;34(3): 353–363. 11902852 10.1006/pmed.2001.0989

[33] Stables GJ, Young EM, Howerton MW, Yaroch AL, Kuester S, Solera MK, et al Small school-based effectiveness trials increase vegetable and fruit consumption among youth. Journal of the American Dietetic Association 2005;105(2): 252–256. 15668684 10.1016/j.jada.2004.11.031

[34] Costanzo PR, Shaw ME. Conformity as a Function of Age Level. Child Development 1966;37(4): 967–975.

[35] Clark RA, Delia JG. The Development of Functional Persuasive Skills in Childhood and Early Adolescence. Child Development 1976;47(4): 1008–1014.

[36] Roberto CA, Pomeranz JL, Fisher JO. The need for public policies to promote healthier food consumption: A comment on Wansink and Chandon (2014). Journal of Consumer Psychology 2014;24(3): 438–445.

[37] Ogden CL, Carroll MD, Kit BK, Flegal KM. Prevalence of childhood and adult obesity in the united states, 2011–2012. JAMA 2014;311(8): 806–814. 10.1001/jama.2014.732 24570244 PMC4770258

[38] van Stralen MM, Yildirim M, te Velde SJ, Brug J, van Mechelen W, Chinapaw MJM. What works in school-based energy balance behaviour interventions and what does not? A systematic review of mediating mechanisms. International Journal of Obesity 2011;35(10): 1251–1265. 10.1038/ijo.2011.68 21487398 PMC3191379

[39] Ebbeling CB, Pawlak DB, Ludwig DS. Childhood obesity: public-health crisis, common sense cure. The Lancet 2002;360(9331): 473–482. 12241736 10.1016/S0140-6736(02)09678-2

[40] Haynos AF, O'Donohue WT. Universal childhood and adolescent obesity prevention programs: Review and critical analysis. Clinical Psychology Review 2012;32(5): 383–399. 10.1016/j.cpr.2011.09.006 22681912

[41] Evans CE, Christian MS, Cleghorn CL, Greenwood DC, Cade JE. Systematic review and meta-analysis of school-based interventions to improve daily fruit and vegetable intake in children aged 5 to 12 y. The American Journal of Clinical Nutrition 2012;96(4): 889–901. 22952187 10.3945/ajcn.111.030270

[42] Reicks M, Redden JP, Mann T, Mykerezi E, Vickers Z. PHotographs in lunch tray compartments and vegetable consumption among children in elementary school cafeterias. JAMA 2012;307(8): 784–785. 10.1001/jama.2012.170 22302602

[43] Just DR, Wansink B. Better School Meals on a Budget: Using Behavioral Economics and Food Psychology to Improve Meal Selection. Choices 2009;24(3): 1–6.

[44] Schwartz J, Riis J, Elbel B, Ariely D. Inviting Consumers To Downsize Fast-Food Portions Significantly Reduces Calorie Consumption. Health Affairs 2012;31(2): 399–407. 10.1377/hlthaff.2011.0224 22323171

[45] Anzman-Frasca S, Savage JS, Marini ME, Fisher JO, Birch LL. Repeated exposure and associative conditioning promote preschool children's liking of vegetables. Appetite 2012;58(2): 543–553. 10.1016/j.appet.2011.11.012 22120062

